# Supplementary material for: Exploring perceptions, challenges, and opportunities of UDS health professionals’ students and faculty regarding community-based education programs for interprofessional education: A study protocol
Source: PLoS One. 2026 Apr 30;21(4):e0330015. doi: 10.1371/journal.pone.0330015 (PMC13132188; doi:10.1371/journal.pone.0330015)
Supplement: Attachment — Submitted filename: Reviewer Response_Plos One.docx [file pone.0330015.s001.docx]

Hello authors,

This protocol addresses an important area in health professions education by proposing to examine community-based education programmes as platforms for interprofessional education. Given that protocols are intended to establish conceptual clarity, methodological coherence, and feasibility prior to study implementation, several aspects of the protocol would benefit from further clarification. Addressing these points at the protocol stage is important for ensuring methodological coherence and strengthening the study’s eventual scholarly contribution.

The following questions are posed to support refinement of the study’s conceptual framing, and the intended contribution of the study.

**Introduction**

1. How do the authors distinguish between “interprofessional” and “interdisciplinary” learning in this study? The manuscript appears to use these terms interchangeably, yet they represent distinct educational constructs with different theoretical and practical implications.
2. On what theoretical framework is interprofessional education grounded in this study?
   While WHO definitions are cited, the introduction would benefit from a clearer articulation of the conceptual or educational theory guiding the interpretation of IPE within community-based contexts.
3. Do the authors conceptualise COBES and TTFPP as inherently interprofessional, or as interdisciplinary programs with potential to support IPE? Clarifying this distinction is important for understanding the study’s underlying assumptions.
4. How explicitly are IPE competencies (e.g., role clarification, interprofessional communication, collaborative decision-making) embedded within COBES and TTFPP?
   The introduction describes collaborative activities, but it is unclear whether these align with established IPE competency frameworks.
5. Is participation across different health professions mandatory, structured, or incidental within COBES and TTFPP? This distinction affects whether the programmes can reasonably be described as interprofessional education.

Research gap and rationale,

6. What specific aspect of the relationship between COBES/TTFPP and IPE is currently unknown? For example, is the gap related to educational outcomes, student perceptions, programme design, implementation processes, or policy relevance?
7. Why does this gap matter for health systems strengthening and health professions education policy? The Introduction will benefit from clearer articulation of the implications if this gap remains unaddressed.
8. Is the identified gap global, national (Ghana), or institutional (UDS-specific)?
   Explicit clarification would strengthen the scope and transferability of the study’s contribution.

Evidence base and justification of claims

9. How did the authors determine that evidence linking COBES and TTFPP to interprofessional education is limited or insufficient? The manuscript would benefit from briefly describing whether a scoping search, narrative review, or consultation of existing reviews informed this claim.
10. Are there existing studies or reviews on community-based interprofessional education in similar country settings that could contextualise this work Referencing such literature would help position the study within the broader evidence base.

Consistency and precision in terminology

11. Please ensure consistent use of terminology throughout the Introduction (e.g., “community-based education,” “community-based interprofessional education,” “interdisciplinary learning”).
12. Some claims regarding benefits of IPE and CBE are broad. Can the authors clarify which claims are empirically supported versus aspirational or policy-driven?

Clarify study focus and contribution

13. What is the primary unit of analysis in this study: educational design, student learning outcomes, or institutional implementation of IPE? Clarifying this would help align the Introduction with the study aims.
14. What is the intended contribution of this study to the IPE literature—conceptual, empirical, or programmatic? Making this clear would strengthen the manuscript’s scholarly positioning.

**Problem Statement**

1. The problem statement appears to treat COBES and TTFPP as equivalent platforms for interprofessional education, despite notable structural differences between the two programs. Given that COBES involves only medical students, while TTFPP engages students from multiple health disciplines, could the authors clarify how COBES is being conceptualised in relation to interprofessional education? Specifically, is the study examining:
   - Existing interprofessional practice,
   - Perceived opportunities for IPE, or
   - Potential for future interprofessional integration within these programs?
     Clarifying the distinction between interdisciplinary exposure and interprofessional learning in this context would strengthen the problem statement.
2. The phrase “lack of studies creating awareness” is vague. Consider replacing it with more precise language, such as, “lack of empirical evidence, “or “lack of systematic evaluation,” or “limited qualitative insight into stakeholder experiences.”
3. The problem statement would be strengthened by linking the identified gap to broader educational and health system outcomes, including, curriculum reform, workforce readiness, and /or health system strengthening.

**Aim, Research Questions & Objectives**

1. Terms such as “health professionals,” “health professional students,” and “faculty” are sometimes conflated. Please ensure consistency and explicit differentiation across the aim, research questions, and objectives.
2. Consider explicitly referencing IPE competencies (e.g., communication, roles/responsibilities, teamwork) to sharpen the analytical focus of the study.
3. While the research questions and objectives outline an exploration of perceptions, challenges, and opportunities, the conceptual framing remains unclear. Specifically:
   - How are structurally different programs (COBES as mono-professional and TTFPP as multi-disciplinary) conceptualised as platforms for IPE?
   - Is the study focusing on existing interprofessional practice, perceived opportunities, or aspirational future integration?
     Clarifying these points would strengthen alignment between the problem statement, research questions, and the study’s stated significance.
4. The main and specific research questions substantially overlap, with the specific questions largely reiterating components of the overarching question. Streamlining or reframing the research questions would improve analytic clarity and alignment at the protocol stage.

**Methodology**

1. Please consider specifying:
   - descriptive vs interpretive phenomenology

The protocol mentions researcher reflexivity but does not clearly specify how potential biases arising from the researcher’s professional background and prior assumptions will be addressed. Given the phenomenological design, the authors could clarify the practical steps they will take to ensure that their preconceptions do not influence data collection, coding, and interpretation. For example, they could describe the use of reflective journals, peer debriefing, audit trails, or member checking to explicitly manage researcher positioning and maintain methodological rigor.

**Study area & Population**

Some geographic details (GPS coordinates) may be unnecessary for a protocol. Please

streamline to focus on *educational relevance* rather than locational precision.

Provides context but does not clarify:

1. How COBES is conceptualised in relation to interprofessional education, given it is mono-professional.
2. Whether the study is examining actual interprofessional practice, perceived opportunities, or future integration of IPE.
3. How interdisciplinary vs interprofessional learning is being distinguished in the study.

**Population**

1. Why include final-year medical students for COBES but only TTFPP for other disciplines?
2. Are first- and second-year TTFPP students excluded? Why?
3. Is the rationale for selecting only final-year students justified?

**Exclusion criteria**

1. Excluding participants who are ill, or absent during data collection may introduce bias. How will the study handle this?
2. What about students/faculty who have participated in one program but not the other?

**Alignment with research aim**

1. Does the inclusion of only final-year students capture the full spectrum of experiences with IPE in these programs?
2. How will the study handle COBES, which is mono-professional, versus TTFPP, which is interprofessional?

**Justification for faculty inclusion**

1. How is “rich experience” operationalized? Are only faculty who have directly supervised IPE included?

**Sampling procedure**

1. Claim that findings will be “generalizable” is methodologically inaccurate.

**Data collection procedures**

1. Please clarify:
   - whether interviews will differ for students vs faculty

**Data Analysis**

“Triangulation” is mentioned, but only one data source is used.

1. Please reframe as *methodological rigor* rather than triangulation or clarify triangulation of perspectives (students vs faculty).
2. Please state how many people will be involved in the thematic analysis?

**Sources of data**

1. First sentence, please write ‘anal’ in full – ‘analysis’.

**Ethical considerations**

1. In the first sentence, please change the word ‘issued’ to ‘sought’.

**Trustworthiness & Reflexivity**

1. A language error in this section (“This study assure trustworthiness”) should be corrected.
2. Please consider explicitly stating researcher positionality (e.g., academic role, institutional affiliation).

**Please add a brief section on Data storage**

While the protocol states that ethical approval will be sought, it does not sufficiently describe data storage and management procedures. Given the use of qualitative data involving human participants, further detail is required regarding how confidentiality will be maintained. Please clarify how data will be stored (e.g., secure, password-protected systems), whether transcripts will be de-identified, how audio files and transcripts will be managed, who will have access to the data, and how participant privacy will be protected in accordance with ethical requirements.

**Expected Results, Discussion & Conclusion**

For a study protocol, the Expected Results, Discussion, and Conclusion sections are overly developed and resemble a completed study. Please condense expected results and focus on plausible thematic domains rather than detailed findings.

Protocols usually describe study objectives, methods, and expected contributions, but extensive “predicted outcomes” may be unnecessary or speculative.

1. Could the authors clarify why these detailed expected findings are included, and how they will inform the study design or interpretation once data are collected?
2. How do these anticipated results align with the specific context of UDS? Are these predictions justified by the study design, or are they mainly extrapolated from international literature?

The narrative sometimes reads like actual findings rather than predictions.

1. Can you please ensure that language clearly distinguishes between anticipated insights and actual results?
2. Also, please reduce discussion and shift emphasis to *potential implications* rather than interpretations.
