# Supplementary material for: Exploring perceptions, challenges, and opportunities of UDS health professionals’ students and faculty regarding community-based education programs for interprofessional education: A study protocol
Source: PLoS One. 2026 Apr 30;21(4):e0330015. doi: 10.1371/journal.pone.0330015 (PMC13132188; doi:10.1371/journal.pone.0330015)
Supplement: Attachment — Submitted filename: Plos One Review Report Dec 2025.docx [file pone.0330015.s002.docx]

**PlOS One Review Jan 2026**

**Exploring Perceptions, Challenges, and Opportunities of UDS Health Professionals' Students and Faculty Regarding Community-Based Education Programs for Interprofessional Education-A study protocol.**

Thank you for the opportunity to review this protocol. This protocol provides an informative overview of the planned study in an interesting and valuable area of research. Some key areas to review and revise in this protocol are described below:

- Please specify whether you plan to use a reporting guideline to inform the reporting of your study?
- A position statement is required to provide background on the researchers and their position in the research.
- Please review for inconsistencies in the formatting of your in-text references throughout the manuscript. For instance, including author’s initials within the in-text reference.
- Try to be consistent with your use of abbreviations e.g. Community-based education (CBE) and University of Development Studies (UDS) and avoid redefining abbreviations in each section that have already been provided earlier in the manuscript.

Please see below for feedback relevant to specific sections of your manuscript:

Abstract

- In the methods, please state the specific qualitative research methodology that will be used. Please also identify the thematic analysis process that will be undertaken.

Introduction

- Please check the first reference (WHO.2010) for an extra full stop.
- In paragraph 2, please review the reference (Green BN & Johnson CD., 2015) as authors’ initials not required here.

Aim & research questions

- You have clearly outlined the research questions and objectives in your manuscript, it would be beneficial to include some examples of the interview questions which you will use, to see how they align with the research questions and objectives you have provided.

Population of the study

- Who will be responsible for recruitment of the participants and what will this process entail?
- What sampling strategies will be used as part of this study? I can see this is mentioned in a later section, you could think about incorporating the information about sampling in this section where you describe the sample you will aim to recruit, in order to cut down on excess words and repetition later.
- As you plan to recruit students and members of the faculty, are you going to set a quota for the number of participants from each of these subgroups (i.e. use of quota sampling)?

Variables of the study

- I’m not sure if this section is necessary, considering this is a qualitative study and variables are more of a feature of quantitative study design. I would recommend thinking about your terminology here or removing this section all together.

Sample size

- You have stated that you will aim to recruit “approximately ten faculty members representing a diverse range of health professions”. Will you aim to recruit a certain number from each health profession to capture a variety of different professions within your sample?

Data collection procedures

- Who will be conducting the interviews with the participants? What is their experience in conducting qualitative interviews and will they pilot any interviews prior to data collection?
- You have mentioned the use of an interview guide; can you provide some examples of interview questions that will be included?
- Please identify who will be responsible for transcribing the interviews and how.
- Please specify whether you will conduct any follow up interviews with participants.

Pre-testing/ pilot testing

- Be careful with using terminology such as “validity and reliability” as this is more relevant to quantitative research.
- How many participants will be involved in the pilot testing of the interviews?
- Try to be consistent with the terminology used (re. pilot testing vs. pre-testing).

Data analysis

- Please add an in-text reference for the Nvivo software that you have referred to.
- You have commented on the use of peer debriefing and member checking to achieve validity and reliability. As with my previous comment, please be careful with using this terminology considering this is a qualitative study. Likewise, further explanation of the member checking process is necessary in this section (for instance, what information will be shared with participants in the “summary”?) to explain the purpose of member checking and how it supports rigour and trustworthiness.
- As part of the coding process, will you have multiple researchers involved in the coding?
- “Triangulation of data will also be done through cross-matching results in different sources of data”. What does this mean, can you explain this?

Sources of data

- Spelling error at the end of the first sentence of this section.

Reference list

- Check for inconsistencies in line spacing between each reference.
