# Supplementary material for: Exploring perceptions, challenges, and opportunities of UDS health professionals’ students and faculty regarding community-based education programs for interprofessional education: A study protocol
Source: PLoS One. 2026 Apr 30;21(4):e0330015. doi: 10.1371/journal.pone.0330015 (PMC13132188; doi:10.1371/journal.pone.0330015)
Supplement: Attachment — Submitted filename: Rebuttal letter to Editors and Reviewers Comments.docx [file pone.0330015.s003.docx]

Editors and Reviewers

Dear Editor and Reviewers,

We sincerely thank you for your careful review and constructive feedback. We have revised the manuscript substantially to enhance conceptual clarity, methodological coherence, and reporting transparency. Below we detail how each comment has been addressed and indicate the relevant sections of the revised manuscript.

Editorial Requirements

PLOS formatting requirements

The manuscript has been revised to align with PLOS ONE style guidance. Title consistency has been verified between the manuscript and submission system.

LaTeX template

The manuscript will be submitted using the PLOS LaTeX template as required.

Funding statement

All funding-related text has been removed from the acknowledgements section. The Funding Statement now reads: “The authors received no specific funding for this work.”

Data availability

The Data Availability Statement has been revised to specify that de-identified qualitative transcripts will be deposited in an appropriate open-access repository upon publication (Section 6.3).

ORCID

The corresponding author’s ORCID iD has been validated in Editorial Manager.

Ethics statement

A full ethics statement has been included in the Methods (Section 2.13) and Declarations (Section 6.1), specifying the approving Institutional Review Board and consent procedures.

Reviewer 1

Comment: Clarification of conceptual framing and intended contribution.

Response: The introduction and research gap sections (1.1–1.3) have been revised to clearly distinguish interdisciplinary exposure from interprofessional education and to justify the conceptual positioning of CBE as a potential but not automatic platform for IPE.

Reviewer 2

Comment: Specify reporting guideline.

Response: The manuscript now states adherence to the Standards for Reporting Qualitative Research (SRQR) (Section 2.0).

Comment: Provide researcher positionality statement.

Response: A dedicated section on researcher reflexivity and positionality has been added (Section 2.12).

Comment: Ensure consistency of in-text references.

Response: All in-text citations have been reviewed and corrected for consistency.

Comment: Clarify sampling and recruitment.

Response: Sampling strategy (purposive maximum variation) is described in Section 2.5. Recruitment procedures and responsibility are detailed in Section 2.6.

Comment: Clarify sample composition across professions.

Response: We specify inclusion of final-year students across medicine, nursing, midwifery, pharmacy, and nutrition, and faculty from diverse disciplines (Section 2.3 and 2.5). Sampling will proceed to data saturation rather than fixed quotas.

Comment: Interview procedures and interviewer experience.

Response: Section 2.7 clarifies that interviews will be conducted by the principal investigator, who is trained in qualitative interviewing.

Comment: Pilot testing terminology.

Response: Terminology has been revised to avoid quantitative constructs such as validity and reliability (Section 2.8).

Comment: Clarify coding process and member checking.

Response: Section 2.10 specifies dual coding of a subset of transcripts. Section 2.11 elaborates the member checking process, including sharing concise interpretive summaries to enhance credibility.

Comment: NVivo citation.

Response: An in-text citation for NVivo has been added in Section 2.10 and included in the reference list.

Comment: Triangulation clarification.

Response: The triangulation statement has been removed to avoid conceptual inconsistency with the interpretive phenomenological approach.

Comment: Reference formatting.

Response: The reference list has been reviewed for spacing and formatting consistency.

We believe the manuscript is now conceptually coherent, methodologically transparent, and aligned with PLOS ONE reporting standards. We appreciate the opportunity to strengthen this work and look forward to your further consideration
