# Supplementary material for: Exploring perceptions, challenges, and opportunities of UDS health professionals’ students and faculty regarding community-based education programs for interprofessional education: A study protocol
Source: PLoS One. 2026 Apr 30;21(4):e0330015. doi: 10.1371/journal.pone.0330015 (PMC13132188; doi:10.1371/journal.pone.0330015)
Supplement: Attachment — Submitted filename: Response to reviewers comments(F).docx [file pone.0330015.s004.docx]

**Manuscript ID:** PONE-D-25-39841R1
**Title:** Exploring Perceptions, Challenges, and Opportunities of UDS Health Professionals' Students and Faculty Regarding Community-Based Education Programs for Interprofessional Education: A Study Protocol

**Response to Academic Editor and Reviewers**

Dear Professor Saravana Kumar and Editorial Team,

We sincerely thank you and the reviewers for your careful evaluation of our manuscript and for the constructive feedback provided. We appreciate the positive assessment of the study’s rationale, methodological rigor, and clarity. We have revised the manuscript accordingly and addressed all comments raised.

A point-by-point response to the reviewers’ comments is presented below.

**Reviewer #1**

**Comment 1:**

Line 76. CBE must be written in full at first mention.

**Response:**
Thank you for this observation. We have revised the manuscript to ensure that the term is written in full at first mention as “community-based education (CBE)” and used consistently throughout the manuscript thereafter.

**Comment 2:**

Line 81. Same issue as above with UDS.

**Response:**
We have corrected this by writing the full name “University for Development Studies (UDS)” at first mention and using the abbreviation consistently in subsequent sections.

**Comment 3:**

Line 82. Similar issues with TTFPP and COBES.

**Response:**
We have revised the manuscript to include the full names at first mention: “Third Trimester Field Practical Programme (TTFPP)” and “Community-Based Education and Service (COBES).” These abbreviations are now used consistently throughout the manuscript.

**Comment 4:**

Line 158. The sentence is abstract and does not explain how the study provides clarity.

**Response:**
We thank the reviewer for this helpful suggestion. The sentence in the significance section has been revised to improve clarity and explicitly state how the study contributes conceptually and practically. The revised sentence now clearly explains the distinction between interdisciplinary exposure and structured interprofessional education (IPE), and how this contributes to both research and practice, particularly in resource-constrained contexts.

**Reviewer #2**

**Comment 1:**

Please include a reference to the SRQR reporting guideline.

**Response:**
We have added the appropriate reference to the Standards for Reporting Qualitative Research (SRQR) guidelines (O’Brien et al., 2014) in the methodology section and included the full citation in the reference list.

**Comment 2:**

Provide a more detailed explanation of the thematic analysis process in Section 2.10.

**Response:**
We appreciate this important suggestion. Section 2.10 (Data analysis) has been revised to provide a clear, step-by-step description of the reflexive thematic analysis process. Each phase of the analysis, including familiarization, coding, theme development, review, and reporting, is now clearly explained to enhance transparency and reproducibility. The role of NVivo software and the use of a second coder to strengthen the trustworthiness of the analysis have also been clarified.

We sincerely thank the Editor and Reviewers for their valuable feedback, which has helped to improve the quality of this manuscript. We hope that the revised version meets the journal’s requirements and is suitable for publication.

Sincerely,
Maxwell Ateni Assibi

assibimaxwell93@gmail.com
(On behalf of all authors)
